# Should biomedical research be like Airbnb?

**DOI:** 10.1371/journal.pbio.2001818

**Published:** 2017-04-07

**Authors:** Vivien R. Bonazzi, Philip E. Bourne

**Affiliations:** National Institutes of Health, Bethesda, Maryland, United States of America

## Abstract

The thesis presented here is that biomedical research is based on the trusted exchange of services. That exchange would be conducted more efficiently if the trusted software platforms to exchange those services, if they exist, were more integrated. While simpler and narrower in scope than the services governing biomedical research, comparison to existing internet-based platforms, like Airbnb, can be informative. We illustrate how the analogy to internet-based platforms works and does not work and introduce The Commons, under active development at the National Institutes of Health (NIH) and elsewhere, as an example of the move towards platforms for research.

At first glance, the idea that biomedical research has any relationship to an accommodation rental service probably seems absurd. Let us explain.

As we worked through a strategy for improved data management and sustainability as part of our work with the National Institutes of Health (NIH), one of us (PEB) was also in the process of making an apartment available through Airbnb. While having a number of satisfactory rental experiences with Airbnb, he had never been a host (the person renting to travelers) before. Hosting further exemplifies what one experiences as a renter—it succeeds because it is a relationship built upon **trust**. Using Google Drive or Github are other simple examples of a relationship built on trust. Using the Airbnb software platform, the renter trusts that the accommodation is going to be as advertised; the host trusts that the person renting is not going to trash their property. Host and renter both trust Airbnb to facilitate and manage the transaction. The software platform upon which Airbnb is based makes every effort to gather as much data on both renters and hosts to maximize the sense of trust. The platform is easy to use, and transactions are inexpensive. The service is far from perfect. Issues have arisen concerning how Airbnb can change neighborhoods in areas with high tourist potential [1] or, indeed, of claims of racial bias by hosts [2]. Nevertheless, something is working, since as of February 2016, Airbnb had 60 million users searching 1.5 million listings in 191 countries, with an average of 500,000 stays per night [3]—all leading to a valuation of US$25 billion. So, what does this have to do with biomedical research?

Airbnb supports a trusted service between providers and consumers of that service. Consider biomedical preclinical and clinical research, in which the trusted service involves the exchange of papers, data, software, reagents, and so on. An author publishes a paper having applied rules of scientific conduct and is a supplier. That paper is read by consumers based on how much they likely trust the work based on what they know about the authors and what journal it was published in, which, in turn, speaks to the presumed quality of the review, who else cited it, and what trusted bloggers and other social media contributors have had to say about it. The same applies to data. Knowing what laboratory the data comes from and having sufficient information about the methods used instills a sense of trust, as does a curated data repository that is well managed. A similar trust-based argument can be made for other resources that can be exchanged, including people. A principal investigator will hire a postdoctoral fellow based on the trust they have that the individual will contribute to the laboratory. That trust comes from not only what they have published but where they have published and what their references, themselves trusted investigators (or not), have had to say.

From the perspective of managing the trusted relationship, what is different between Airbnb and biomedical research? Airbnb operates through a single platform through which services are exchanged and transactions conducted; biomedical research has multiple discrete and poorly connected platforms (Fig 1) or no platform at all.

**Fig 1:**
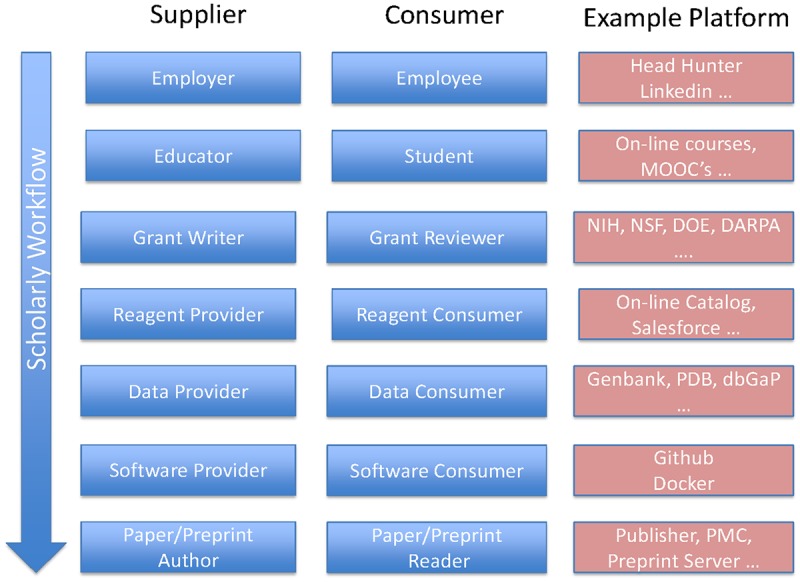
Discrete suppliers and consumers and associated platforms supporting the scholarly workflow. While presented as a linear path for simplicity, in reality, it is more a network of interactions.

Of course, the services being exchanged by Airbnb are much simpler and narrower in scope than those of biomedical research. Biomedical research is akin to the complete travel experience—scheduling, airfares, ground transportation, tours, etc.—not just nightly accommodation. Airbnb also only supports direct services between two distinct parties, whereas biomedical research involves many participants in the exchange. Moreover, Airbnb was born digital, whereas biomedical research has a long analog history, and for many stakeholders, the manuscript still represents the medium through which to communicate research—journals are **the** platform unifying contemporary research. Thus, cultural change is the biggest impediment to a new, presumed value proposition. Airbnb overcame this need for a cultural change by immediately offering a platform and associated trusted service that was a win for all stakeholders. Can the same happen in biomedical research? Probably not, since there are technical barriers greater than Airbnb faced, most notably a smaller, more conservative group of stakeholders and the sheer size of the software development undertaking to create a single, robust platform that is modular and customizable to different stakeholder needs. Nevertheless, niche platforms exist, and their adoption continues to grow. Therefore, we have seen some aggregation between the rows shown in Fig 1, but as yet, a single platform does not come close to encompassing all trusted relationships between consumer and supplier. Rather, they remain a curiosity yet to achieve mainstream adoption. Will that change? It remains an open question.

Consider examples in which the merger of the rows in Fig 1—the traditional boundaries of research—into a single platform would advance the biomedical research enterprise. Realistically, a single platform may be unobtainable as a result of remaining different business and scientific practices, governance models, and technologies. Not to mention the significant heterogeneity of content in biomedical research. Accommodations are easily quantified with a few parameters; biomedical research is definitely not. In that case, some level of interoperability between rows may be more realistic, but we should aspire to what appears to be a single platform, even if it is not.

Publishers provide an exchange of services between an author and readers; data resources provide an exchange of services between a data provider and a data consumer; software developers provide software to a user community. Think about these exchanges from the point of view of first, the transaction that takes place and second, the platform used to make the exchange. The transactions are similar, but the platforms are not, as one of us wrote 11 years ago [4]. Regrettably, not enough has changed in the past 11 years, even though the technology to support that change is available. What is still lacking is broad adoption through new business models and incentive structures. Consider examples of the nature of these exchanges.

The authors submit a paper to a journal they trust via an editor who seeks reviewers who pass judgment on the paper, usually without review of any data or software used to generate the results and the conclusions presented. Assuming the paper is perceived to make a worthy contribution, one or more iterations occur between authors, reviewers, and editors before the paper is published and appears online. That paper is then indexed by PubMed, Google Scholar, etc. so that it can be found and the findings disseminated. There are a small number of commercial platforms used by publishers to process manuscripts, garner reviews, and make available the final article in a small number of formats, notably HTML, PDF, and XML (particularly JATS). The form of the output—abstract, introduction, material and methods, and so on—is consistent across the corpus, but the end product is disjoint from the data, software, materials, etc. used to support the findings. This was a natural consequence of an analog world where these were physically disparate entities, but it makes no sense in a digital world where even physical entities, e.g., reagents, instruments, have a digital signature that uniquely defines and describes the resource.

Data provision and use follows a similar process **yet is completely disjoint from the publishing process in most research domains**. The data generator, under the auspices of the research funder and the data generator’s institution, both with conditions for data sharing, provides the data to an identified trusted repository. That submission is reviewed, either automatically or manually by biocurators or in combination. At which point, the submission is accepted or returned to the data producers for improvement. Once accepted, these data are provided with a unique identifier and in a format typically defined by the field of researchers who generate this type of data. So far, the process for the exchange of data is identical to the exchange of knowledge embodied in a paper. As such, it makes no sense to separate the two, particularly when a serious understanding of the paper, as for example, in reproducing the results, requires that the data and analytical methods, likely embodied in software, be accessible. Alarmingly, post-publication and data and software deposition, huge amounts of time and resources are spent by researchers essentially putting back together the various pieces of the research process that were taken apart in the name of analog-based dissemination.

The senselessness does not end there. Whereas at least research papers are presented somewhat uniformly, data are not. In fact, the means by which data infrastructure is funded, typically as part of a research endeavor, fosters environments that seek to be as different to each other as possible so as to highlight their advantages. The end result is a multitude of different user interfaces, access methods, and general lack of uniformity—something funders should collectively address. Contrast this to Airbnb in which a single easy-to-use platform manages all transactions, for example, local taxes and payment from renter to host are all are handled uniformly. To anticipate that all funders would use a single platform for transactions between suppliers (the agency) and the consumer (awardees) is unrealistic, yet working together to create a more uniform supplier side as it relates to data and other resources is where progress can be made. For example, the Food and Drug Administration (FDA) has taken steps to make things more uniform—all trials that the FDA regulates have to sit on a Clinical Data Interchange Standards Consortium (CDISC) platform. This will make it easy for the FDA to compare trial results to each other. Similarly, the NIH is encouraging more uniform use of common data elements across the Institutes and Centers.

A final act of inefficiency occurs when important data that were available in digital form, but presumably had no obvious data repository as a home, are later manually extracted from the paper and placed into a digital repository at significant cost. The value of a platform that integrates data, software, and research articles in a way that is trusted by suppliers and consumers should be obvious.

While beyond the scope here, the change that platform adoption, in the way described above, could have on scholarly communication is enormous. Suddenly the unit or currency is not the artificial boundaries imposed by journal article page limitations and the amount of supplemental data that can be included in a paper but becomes a matter of attributable units of data and associated narrative that could change how we conduct and report scientific research.

So far, we have focused on the relationship between the data and publication layers of the scholarly workflow, yet other layers of the research enterprise, as embodied in Fig 1, can also be described in a way that points towards the need for greater integration. Software is increasingly available through open platforms like Github, which is commendable, but again usually disjoint from the research to which it was applied, although at least links to software are beginning to appear in research articles and elsewhere.

The commercial world has its own platform technology broadly referred to as Customer Relationship Management (CRM), exemplified by products like Salesforce. CRMs manage the interactions between a customer and a supplier and have some limited use in biomedical research, but there is nothing substantive for managing that trusted interaction that happens around, say, the exchange of antibodies, exchange of best practices for a given experimental protocol, and many other exchanges that form the basis of biomedical research. Such exchange is ad hoc. You discover reference to an antibody in a product catalog or reading someone’s paper. Best practices are only exchanged years after they could be after you discover, through the literature or a conference, that a laboratory is adept at a procedure, and you send a student to learn it. Moreover, CRM’s are disjoint from other aspects of the research enterprise, such as publishing platforms, data archives, software archives, and so on.

Similar arguments can be made regarding teaching and mentoring. The value of one-on-one mentoring or face time in class should not be disputed. What should be disputed is how best to determine the one to mentor or how to locate the best material to be used in teaching. Software platforms can certainly help in this regard.

In summary, there is not currently a widely adopted single platform or highly integrated platforms for the exchange of services in biomedical research. Either there is a platform per service, or limited set of services, or no platform at all. Why have we not done better, and what are the impediments?

Surrounding the analog model of biomedical research is a strong tradition and a set of business practices that drive the enterprise. Change to such an ecosystem generally comes slowly. From a business point of view, such a change can involve a very significant investment without a clear understanding of the value of that change to the business in question. Only when the customers see the clear value of making a change is it likely to drive that business into making a change. This is antithetical to the attributed Henry Ford approach of, “If I had asked the people what they wanted, they would have said faster horses,” or Steve Jobs for that matter, “A lot of times, people don't know what they want until you show it to them.” These giants had two advantages in overcoming the inability of stakeholders to see the value—technology and money. The production line for the Ford Model T is an example of technology and, well, Apple has a valuation greater than the gross national product (GNP) of two-thirds of the world’s countries, which speaks to having money.

Is there a Ford, an Apple, or a philanthropist for that matter, willing to make the investment in developing a platform for biomedical research that embraces all the needed exchanges? It would seem that the answer is no. Certainly, in academia there is neither the incentives—it won’t bring you tenure—nor long-term funding to undertake such an endeavor. Rather, we have a situation where government funders, foundations, and the private sector have each invested in a small piece of what is needed (Fig 2). It is conceivable that one or more commercial entities could become the platform provider. Some publishers are indeed moving in this direction to satisfy a vertical market through a larger part of the scholarly workflow. There would certainly be resistance by some to this commercialization. Moreover, what is required to succeed would seem beyond the capabilities of any company, and an open and collaborative system built by scientists for scientists would seem to have the most likelihood of success.

**Fig 2:**
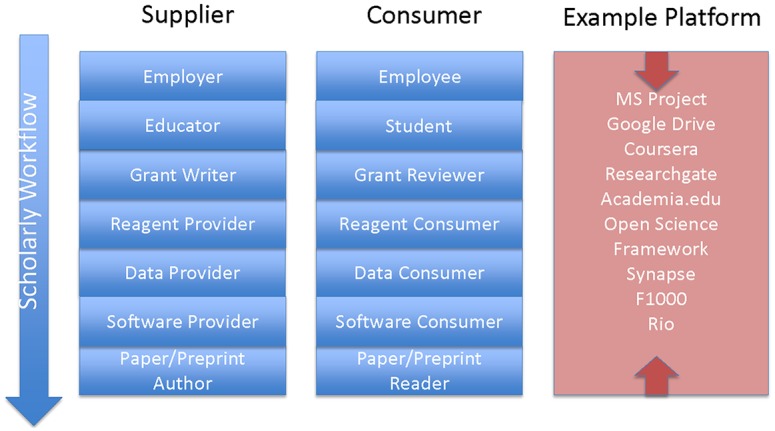
Example platforms currently supporting biomedical research.

Fig 2 by no means covers all platforms in use in biomedical research, and each of these example platforms addresses a varying amount of the biomedical research enterprise. For example, Google Drive enables file sharing in a generic way and thus facilitates the exchange of data, software, and knowledge but does not directly support actions on these particular objects of research nor has a mechanism to instill trust. Synapse [5], in a specialized way, and the Open Science Framework (OSF) [6], in a more generic way, both support the exchange of these research objects and actions upon them. Researchgate [7] supports the exchange of knowledge through papers and social networking between people. Coursera [8] supports a learning environment but no other forms of exchange. Each is a piece of the puzzle but not a broad solution. OSF comes close to being a generic exchange platform with the ability to plugin what the community requires as the system evolves, thus fostering a uniform digital ecosystem. Not only are current platforms not a broad solution, they currently occupy small, niche markets. Their ability to scale and adoption at scale remains questionable.

What would be the advantage of having a more complete trusted exchange platform across the research enterprise? Consider a couple of hypothetical use cases.

An obvious example is the long talked about executable paper. Rather than the paper being an advertisement for the research [9] it becomes an actionable entity. Computational components of the analysis can immediately be rerun, data associated with the study explored, and modifications made to the experimental protocol and the experiments rerun, perhaps on large amounts of data in a cloud environment. As such, this truly builds on the original study. This of course happens now, as any graduate student could attest, but in an arduous, time-consuming, and hence costly way. You download what you can by way of data and software associated with the paper and attempt to recreate and build upon the experiment. We attempted to quantify the added cost associated with these necessary actions for one in silico experiment [10] and came up with 280 hr of added labor 3 yr after the original work was carried out. Now, 6 yr after the original experiment was conducted, the cost of reproducing or building on the work would be much higher, if indeed it was possible at all, as data and software atrophy in a project that no longer has grant support.

A slightly less obvious example is the communication between producers of research and the people who stand to gain the most from those products and from each other. At present, that relationship is limited and disjoint. You may receive alerts of work from a particular laboratory, mostly through the papers they publish. This is not timely or relative to when the work was carried out and, in our experience, these are usually inaccurate. As the consumer of that research you end up with useless interruptions, and the producers of that research have no way of knowing you are interested in their work. Tweets and other social media provide a blanket awareness but there is no accurate, ubiquitous, trusted point-to-point interaction. That interaction can come from matching an accurate and current profile of the scientist wanting to be alerted to metadata and/or the accurate extraction of semantic content from the paper, Github, wikis, Twitter, etc. The technical wherewithal to make this happen exists, but accessibility on the same trusted platform is prerequisite and does not exist in a broad context. Broadly speaking, the problem is the social contract needed to make this a reality.

A social contract can potentially develop over time, provided the platform is in place and parties to the contract have a measure of trust in each other. Even then, incentives are needed. In the case of Airbnb, the platform was enough of an incentive even though other similar services existed. Why Airbnb would become the dominant platform has been discussed most notably by Sangeet Paul Choudry [11]. In the case of biomedical research, there is already a way to conduct business as usual, so further incentives are needed.

Scientists themselves are incentivizing others, for example, the work of Ben Best as Duke University using cloud-based data services with R code executed directly from Github [12] or those posting their Jupyter [13] notebooks. However, this is not enough to incentivize the whole community in a significant and timely way.

Funders and publishers are uniquely positioned to provide those incentives. It is not clear that traditional publishers will do so—they have too much to lose from their current business practices. If they are trying to do so, it is not clear they will succeed as described above. Newer nontraditional publishers have the desire to change the status quo but not the resources and/or level of trust from the community to make a difference.

Thus, it falls to funders to change the system. They should certainly be incentivized to do so given the current questions being raised about the reproducibility of science. Platforms would seem to be one means of simplify the testing of reproducible results. However, there are processes and procedures that one can think of as the equivalent of business practices that are not easily changed to enable the wholesale adoption of a biomedical platform. At NIH, we are taking an agile approach and creating the Commons [14] as our platform. By Commons, we mean an open and inclusive storage and compute environment, which facilitates the discovery, execution, and reuse of digital research products. The Commons constantly builds upon itself as new research products are generated, retained in the Commons, and reused by the broad community of users. By agile, we mean running a series of pilots to evaluate the value of a large-scale effort. Those first experiments use public infrastructure, notably clouds, to support the platform, which is focusing on computing across large datasets. Initially, there is not a focus on people, educational materials, digital signatures for reagents, and the like (Fig 2); if successful, that will come later.

What can we learn and evaluate from such a limited Commons experiment? An obvious potential benefit is that there is no other option than the cloud for computing on large datasets. Less obvious is the trusted sharing of data, software, and computational protocols implied by the Commons. In principle, as new data and methods are generated from the initial experiments, they too are accessible on the Commons platform. For this to work, users must be able to find the content on the platform, and they must be able to trust it (or not). To be findable, content must be indexed, and that requires that each component of the research be uniquely identifiable and resolvable. Various experiments underway through the NIH Big Data to Knowledge (BD2K) program, notably DataMed [15], provide indexing tools, the metadata, and associated standards to make this workable. Broad access and usage of the research components resident on the platform at least provide some metric for the trust that users have in those components, and more elaborate incentives provided through the platform can be envisaged. We may also learn that the concept of a software platform in the form of a Commons does not work.

Airbnb incentivizes suppliers and consumers to provide the best service through alerts, comparisons to those providing similar services, and so on. A similar model can be imagined for research in which, for example, comparative usage metrics and recommendation engines for data and software can speak to the relative value of respective services. The Commons is evolving to potentially be that service. Unlike Airbnb, however, it is not a closed and proprietary system but rather a system built on openness. Scientists are being incentivized to participate in the Commons, either through the provision of large datasets for which compute must be taken to the data or through the provision of Commons credits. Commons credits are dollar denominated and requested by investigators. An investigator awarded credits can spend them with any **Commons-compliant** provider. Commons compliance implies a mild form of regulation and governance, which could be expanded over time if desired by all stakeholders in the Commons effort. To date, a number of public cloud providers and software as a service (SaaS) providers have signed up and agreed to be Commons compliant. Investigators can choose where to spend Commons credits, driving competition into the marketplace. A potential longer-term advantage of Commons credits is the efficiency of a pay-as-you-go business model. The NIH only pays for compute services used and provides a way of matching supply to demand. This differs from the current grant funding model in which resources are provided up front, whether they are fully utilized or not. Another potential advantage of working with Commons compliance providers is the ability to index Commons content and gauge its usage, offering the potential for more informed decisions about what to continue to fund, based on community usage.

Funding alone will likely not be enough of an incentive, even if the Commons credits pilot proves successful—there must also be a scientific advantage. The potential of FAIR [16]—Findable, Accessible, Interoperable, and Reusable—scientific content will help, for inherent in the model is a reliance on standards. Moreover, if implemented correctly, the provider of scientific content will gain from metrics that describe how much that content is used and also have an easy pathway to meeting the sharing requirements from funders. Users of content will be able to find and use relevant content. The system at large will gain by improved efficiency, reproducibility, and the potential for new discoveries: efficiency, as there will be less need to reinvent the experiment—even those from your own laboratory; reproducibility, by making available the complete workflow; and new discoveries, from the aggregation of multiple types of data. As the contents of the Commons moves from data and analytics to include, grant materials, narrative, educational materials, and other aspects of the research lifecycle, the promise of a single platform could be reached.

More concretely, we hope to have some sense of the value of the Commons as a platform in a year or two, based on pilots that are underway or soon to start. Success would see the services offered extend into additional layers of the scholarly workflow (Fig 2). Just as Airbnb has created a new and frequently rewarding way to arrange accommodation, we hope for a more cost-effective and productive way to perform biomedical research, even as the scope and complexity of the two marketplaces is so different.

In summary, coming back to the question posed by this commentary, should biomedical research be like Airbnb? The question remains open. Part of Airbnb’s success and other platform services, for example, Uber and eBay, is the utilization of underutilized capital—spare rooms, car and drivers, unwanted goods. Do research laboratories have underutilized capital? Possibly, but they do not necessarily see the need to exploit, for example, raw data, small software scripts, variations on published protocols, etc. Should that change? Again, unresolved, but the likening to Airbnb is a useful metaphor for extending the debate.

## Abbreviations

BD2K: Big Data to Knowledge
CDISC: Clinical Data Interchange Standards Consortium
FAIR: Findable, Accessible, Interoperable, and Reusable
FDA: the Food and Drug Administration
GNP: gross national product
NIH: National Institutes of Health
OSF: Open Science Framework
SaaS: software as a service
